# AAV Capsid Modification and Its Influence on Viral Protein Stoichiometry and Packaging Fitness: Current Understandings and Future Direction

**DOI:** 10.1007/s12033-025-01381-0

**Published:** 2025-01-29

**Authors:** Dennis Makafui Dogbey, Stefan Barth

**Affiliations:** 1 Medical Biotechnology and Immunotherapy Research Unit, Institute of Infectious Disease and Molecular Medicine, Faculty of Health Sciences, University of Cape Town, Cape Town, 7700 South Africa; 2 South African Research Chair in Cancer Biotechnology, Department of Integrative Biomedical Sciences, Faculty of Health Sciences, University of Cape Town, Cape Town, 7700 South Africa

**Keywords:** Adeno-associated virus, Stoichiometry, Genetic modification, Mutagenesis, Viral protein

## Abstract

The field of gene therapy has witnessed significant advancements in the utilization of Adeno-associated virus (AAV) owing to its inherent biological advantages. Targeted AAV vectors are generated through genetic or chemical modification of the capsid for user-directed purposes. However, this process can result in imbalances in viral protein sequence homogeneity, stoichiometry, and functional transduction vector units, thereby introducing new challenges. This mini review focuses on the ongoing efforts to develop targeted vectors, which inadvertently present unsolicited obstacles for clinical application and provided perspectives on future directions.

## Introduction

Gene therapy is a unique medical treatment modality which involves the modification of the genetic structure of the recipient's genome through the deletion, insertion, or replacement of specific nucleic acids in diseased cells. Its global impact extends across various disease indications with the potential to cure some of the world's deadliest diseases [1, 2]. Of all gene therapy delivery vectors, AAV is currently the leading delivery vehicles for the therapy of a range of diseases. However, preclinical and clinical studies of AAV-based gene therapies are limited by antagonistic immunogenic responses driven by the host, requirement of higher vector doses, off-target toxicities and limited tissue transduction. This is solely attributed to the promiscuity of the capsid towards a diverse range of tissues and its seroprevalence. Because of these major limitations, the design of targeted AAV vectors to selectively target tissues of choice has been based on engineering different capsid protein and insertion of unnatural amino acids. In this context, capsid modification has been conducted to generate recombinant variants with new potentials. Since AAV utilise its capsid protein to interact with the host cell, it therefore remains the integral part for cell targeting and mediating immunotoxicities by AAV-based gene delivery vectors [3].

**Fig. 1:**
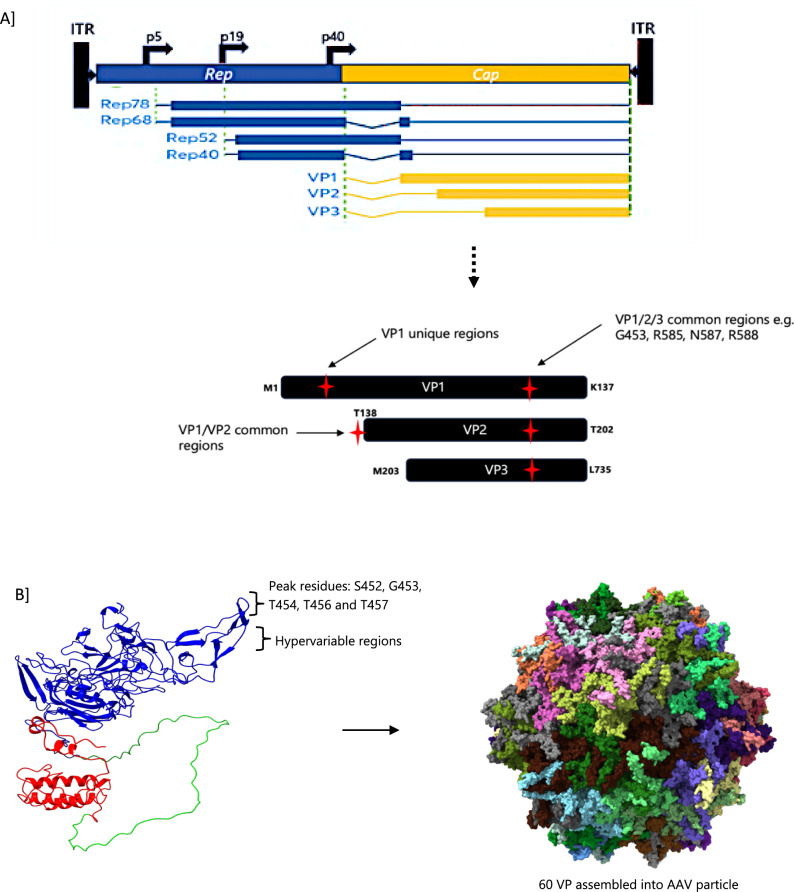
An illustration of AAV capsid protein common modification sites (**A**) and representation of capsid structure showing a monomer (VP1-red, VP2-green and VP3-blue) and the assembled 60 monomers (**B**) (PDB ID 5IPI). The individual VPs forms the capsid structure which is driven by the native p40 promoter. The assembled 60 monomers comprise of −5-VP1, 5-VP2 and 50-VP3 protein units VP1 is within VP2, while VP3 forms part of VP2 and VP3. The hypervariable regions within the surface exposed residues of VP3 dictate the differences between AAV serotypes. All VPs have the same C-terminus. Structures were modelled by using AlphaFold prediction tool

AAV capsid consist of three distinct viral proteins (VP1, VP2, VP3) in the ratios of 1:1:10 (Fig. 1). Manipulation of one or more of the VPs includes the introduction of ligands, and scaffolds to increase tissue specificity, increase packaging yields, hide the vectors from provoking an anti-AAV immune response, among others [4–6]. Capsid modification has been shown to substantially improve transduction activities of AAV vectors thereby reducing anti-host immune responses [7]. Similarly, Mao et al., demonstrated that the single point mutations T251A and S503A in the AAV-DJ capsid significantly improved expression of dual-luciferase in in vivo models which can be attributable to an extended retention time and transgene distribution [8]. Recombinant AAVs have also been shown to persist in tissues primarily as extrachromosomal elements, raising concerns about whether VP modifications which dictate cell transduction, can enhance capsid persistence in targeted cells [9, 10]

### Sites for Insertion and Stoichiometry of AAV VPs

Site specific installation of antigen-specific ligands is mostly preferred to quantify the vector outcome. However, the scale of modifications to the capsid with or without disrupting its assembly is influenced by the site of integration and size of the inserted ligand. It is also an influencing factor on the stoichiometry of individual VPs per a capsid. For example, insertion of scaffolds on VP1 unique domain for affinity conjugation have shown unpredictable capsid structure and assembly [11], in comparison to modification of the VP1/VP2 overlap regions with non-cognate peptides which have resulted in chimeric AAV vectors which retained comparable transduction properties and high vector genome copy numbers [12]. On the contrary, insertion at the N-terminus of VP2 have resulted in reduced tumour cell transduction compared to the unmodified vectors [13]. Because VP1 is the most abundant capsid protein and harbours critical motifs required for viral infectivity and capsid assembly, insertion permissibility (specifically within the VP1 unique region) must be well informed by computer-guided simulation studies [14]. For example, the insertion of a small RGD ligand at VP1 unique region (sites 46 and 115) yielding stoichiometry of 5 resulted in vector instability and fragmentation of the AAV capsid [15]. In contrast, insertion of a ligand at VP1/VP2 and VP1/VP2/VP3 common regions generate stoichiometry of 10 and 60, respectively (Fig. 1). Thus, it appears that irrespective of the site of integration, substantive evidence shows an inverse correlation between large-size ligands, the site of insertion, packaging fitness and altered stoichiometric ratios of the resulting vector.

Intentions to achieving high stoichiometry of capsid VP for increasing the exposure of installed scaffolds for affinity conjugations is highly complex. In this regard, it requires striking a delicate balance between receptor or affinity recognition and achieving a high number of fully assembled stable virus genome copies. The current AAV production processes have been aiming towards improving vector yield. However, the administration of high copy number vectors having poor transduction potentials only contributes to off-target accumulation and immunotoxicities. Instead, a focus on generation of vectors with homogenous capsid proteins (specifically VP1 and VP2) with an improved transduction capacities and stoichiometry. This is because while VP3 is responsible for most of the capsid structure, VP1 and VP2 are involved in target cell transduction and endosomal trafficking and escape [16].

Thus, insertion beyond residue *Met203* (VP3 start codon) falls within the common/overlap regions of VP1/VP2/VP3, making it likely to appear on all 60 capsid monomers. For example, Boucas and colleagues inserted *α*_V_*β*_3_ integrin-specific RGD ligands at site G453, resulting in poor receptor-dependent transduction of approximately 30% and a low yield. In comparison, insertion of GE11 peptide at site R587 yielded higher genome copy numbers but lower transduction capacities. [17, 18]. Overall, it remains unclear whether site modification of an exposed loop or protrusion spikes towards the generation of highly decorated capsid variants or insertion at a less significant terminus is ideal towards the development of targeted AAVs [19]. Modifying by point mutation at (or near) the primary antigen binding motifs (arginine-rich region 561–588 [20] seems to have satisfied most of the above conditions in some cases. These include 1] protruding residues which are surface exposed required in host cell interactions, 2] mutations may lead to ablation of native antigen binding and 3] retention of biology and capsid stability as previously reported. Unfortunately, substitution of N587 with a large RGD tripeptide protein for selective infectivity of brain tumours overexpressing high levels of $\alpha$_v_*β*_3_ integrins receptors resulted in truncated viral particles without VP1 and 2 viral proteins [21]. Thus, to avoid the aforementioned challenges, random modifications targeting capsid surface-exposed lysine residues have also been reported. Recently, Mulcrone and colleagues chemically modified capsid surfaces with N-ethylmaleimide molecules which covalently bonded to lysine residues for the targeting of CD31, CD34, and CD90 expressing cells in the bone marrow of mice [22]. This random labelling of AAV capsids has implications for vector dosage calculation, as the number of labels per vector varies.

### Types of Antigen-Specific Ligands

Another important aspect of AAV capsid modification is the type of antigen targeting moiety for decorating capsid proteins. Currently, the deployment of AAVs as a vehicle for gene delivery is dependent on serotype specific tissue tropism. Therefore, targeted vectors incorporating receptor-specific ligands are constructed to recognise specific diseased target. For example, AAV8 readily exhibited tropism towards mouse retina in preclinical models [23, 24], AAV2 and 4 shows the highest transduction of the photoreceptors of the eye compared to other serotypes [25] and same for AAV2 [26]. Also, AAV2 and 6 has been shown to transduce tissues of mouse airways while accumulating in other organs [27]. These studies highlight the stochasticity and high heterogeneity of AAV capsid based on the serotype dependent interaction of the primary receptors. The native receptor for AAV serotypes 1 and 6 is sialic acid [28], AAV serotypes 2, 4, 5 is heparan sulphate proteoglycan (HSPG), that of AAV9 is shown to be Terminal N-linked galactose glycans in a desialylated Chinese hamster ovary (CHO) cell [29]. Moreover, there are a handful of secondary receptors that co-mediate transduction and infectivity by AAVs [30]. Therefore, the next generation of recombinant AAV vectors for gene therapy would not only benefit from emerging genome engineering technologies and novel synthetic promoters but also engineered capsid as the second most important component of AAV particles [31]. Similarly, gene transduction capacity of capsid modified AAVs varies among different serotypes. Therefore, the selection of the appropriate AAV serotype relies on tissue-specific transduction and its efficiency [32]. Table 1 is listing selected examples of capsid modification strategies of antigen-specific ligands inserted on AAV capsid that were found in the literature.

**Table 1:** Examples of AAV capsid modifications

Ligand inserted	Sites (serotype)	Purpose	Outcome	References
Peptide library derived MLIV.K and MLIV.A capsids	N587 (AAV2)	For improved liver transduction and gene delivery	Off-target accumulation in the spleen and the heart	[42]
Chemokine binding domain of rat fractalkine (CX3CL1) and leptin	T138 (AAV2)	To re-direct tissue tropism	VP2 translation initiation site can tolerate the insertion of large peptide	[19]
Human proprotein-convertase subtilisin/kexin type 9 (PCSK9) derivative	T491-S501 N510-D514 (AAV2)	To direct tropism towards fibroblast activation protein (FAP) and programmed death-ligand 1 (PD-L1) receptors	Diversification capsid tropism	[39]
Short liver-trophic peptides	Q574 + 1 (AAV5)	To improve liver-directed transduction	Vectors exhibited propound post-translational modification	[37]
Lys-Lys	variable loop VIII (AAV2 and AAV5)	To generate vectors with lung-specific tropism	Poor production yield-comparatively poor infectivity	[43]
IgG binding domain of protein A (Z34C)	N587 (AAV2)	To transduce distinct human hematopoietic cell	Poor yield	[44]
GE11	R585 to R588 (AAV2)	To target EGFR-overexpressing tumour cells	Low packaging efficiency	[18]
ASSLNIA peptide	R587 and R588 (AAV2)	A muscle-targeting peptide towards striated muscles on systemic delivery	Ablated heparin-binding motifs. Poor infectivity capacity of virus particles	[45]
Displaying designed ankyrin repeat proteins	T138 (AAV2)	Generation of intravenously delivered AAV	Low yield	[34]
3,3′-dithiobis (sulfosuccinimidyl propionate) (DTSSP)	Random (AAV2, AAV8 and AAV9)	Cross linking vectors for altering and localized gene delivery	Poor yield	[46]
Azides- cyclic-RGD	T454, G453, D327, R587 and R588 (AAV2)	Attachment of a cyclic-RGD peptide onto the capsid to target *α*_v_*β*_3_ integrin receptors	Lack of specific site for insertions of chemical handles	[47]

#### Antigen-Binding Polypeptides

Polypeptides with affinity to cell surface receptors have been utilized to modify capsid proteins to minimize the risks of off-target organ transduction by recombinant AAVs which are prone to tissue indiscrimination associated with wild-type capsids [33]. Installation of peptides on AAV capsid surface may allow for systemic and loco-regional delivery of AAV therapies [34]. In this context, modification of capsid VPs by point mutation of residue T138 with polyhistidine-tagged DARPins, a 14 kDa polypeptide were among the very first AAVs with peptide decorated capsid that were reported [34]. Subsequently, targeted delivery of capsid modified rAAV to specific diseased cells have included knocking out of native binding motifs of AAV2 by replacing those important residues with EGFR-specific peptide (GE11) [18]. In this study, the gene sequence for GE11 peptide was incorporated at the exposed N587 loop region on the capsid surface. This modification redirected binding and transduction of the viral particles towards EGFR-overexpressing cells. Also, by installation of a tripeptide *Arg-Gly-Asp* (RGD)-containing peptide at several epitopes of AAV capsid, recombinant viral capsid transduced and delivered transgene independent of their primary receptors [15]. Admittedly, there is unequal seroprevalence of AAV serotypes within different human populations. For example, previous studies shows that AAV5 were absent or have lowest seroprevalence, weakly induced anti-AAV neutralising antibodies compared to others and poorly transduced and infected liver cells in different geographical populations [35, 36]. Based on this premise, a novel AAV5 mutant containing a 7-mer oligopeptide “FAPTPGP” was inserted post Q574 residue on the variable region VIII demonstrated improved gene delivery to the liver compared to other organs [37].

#### Antibodies

Capsid modification with recombinant antibody fragments holds several promising options for trafficking genetic payload into host target cells. In this regard, the redirection of tropism is not only realised, but several other advantages including (1). Insertion of humanised antibodies thereby eventually limiting the risk of generating anti-AAV immune responses. (2) Target specificity for host cell attachment and internalization and (4). Genetic installation rather than post-translational modifications. Successful insertion of larger-sized antibody fragments for the generation of function viral particles may be possible through both rational engineering and random site directed mutagenesis [38]. For example, Kuklik and colleagues genetically encoded two antigen-specific antibodies (anti-fibroblast activation protein and anti-programmed cell death-ligand) by eliminating α_5_β_1_ integrin (N511–513) and HSPG (581 to 589) receptor binding motifs from the capsid proteins, respectively, resulting in bi-specific vectors [39].

#### Others Antigen-Specific Ligands

Other non-antibody scaffolds like affibodies or affimers among others have been used to modify AAV capsid. For example, Warrington and colleagues inserted and compared the coding sequences for the 8 kDa chemokine binding domain of rat fractalkine (CX3CL1), the 18 kDa human hormone leptin, and the 30 kDa green fluorescent protein (GFP) at N-terminus of VP2. The latter was used to track intracellular trafficking and localisation of AAV particles. However, insertion at VP2 and 3 common regions resulted in loss of viral particles due to no expression of VP3 [19]. Recently, Hoffman and colleagues reported the insertion of SNAP-tag on VP1 of AAV capsid. The resulting vectors qualify for conjugation to benzylguanine-modified substrates. Similarly, the insertion of a non-canonical amino acid, Nε−2-azideoethyloxycarbonyl-L-lysine (NAEK) across variable regions (VRs) III to VIII generating 20 NAEK-AAV5 resulted in destabilized capsid assembly and packaging yield [40]. It can be deduced that, although VRs have been shown to have tolerated non-native ligands, there is a likelihood of skewing VP stoichiometry due to the fact that the residues representing VRs are intrinsic to all the 60 capsid proteins. Thus, the disruption of packaging fitness resulting from shifts in the structural disarrangement of the capsid and non-canonical gene sequences, is highly influenced by the adaptive theory of code evolution [41]. Table 1 list the examples of capsid modification with ligands and their insertion sites on the AAV capsid.

## Conclusion

Practically, the stoichiometry of viral particles (VPs) can vary and be imprecise after packaging, deviating from the theoretical ratios of 1:1:10, which is evident even in unmodified capsid proteins [48]. Therefore, increasing the stoichiometry of inserted scaffolds may not necessarily result in uniform vectors of the same phenotype. Homogeneity in the VPs containing the cognate scaffold or antigen-specific ligands contributing to the transduction potentials of the final vector is important. Similarly, since clinical dose calculations of vectors depend on genome copy number, modification strategies that lead to heterogeneous vectors may have direct implications on transduction activities. Additionally, the level of exposure of the inserted ligand or scaffold plays a crucial role in its interaction with the desired receptor or its reaction with conjugating partners, respectively. Consequently, inserting at residues that are not exposed on the capsid surface is unlikely to achieve the aforementioned objective. Lastly, maintaining vector biology and achieving identical vector yield are vital for dose calculations in downstream applications [49]. This requirement is of maximum consideration for industrial biomanufacturing scaling-up processes and eventual clinical applications.

## Outlook and Recommendations

Because delivering functional genetic material using AAV is challenging, the solutions to these issues must be traced back to their origins and addressed technically. Currently, the FDA in the United States has approved over thirty cell and gene products, six of which are AAV-based, with over 200 undergoing clinical evaluations [50]. Majority of these discrepancies revolve around the inability of upstream production processes to overcome challenges related to immunogenicity and off-target accumulation of the generated vectors and the high imbalance between full and empty capsid vectors impacting transgene expression [51]. Recent studies also associated the lifespan of gene expression to the level of gene deposition and integration into the host cell. Collectively, these findings are linking the capsid-dependent transduction of target cell to the expression and functionality of the transgene to be delivered [52]. With regards to engineering AAV, the most reliable solution lies in developing chimeric/synthetic capsids or modifying the naturally occurring capsid sequences to achieve the above-mentioned objectives. Assuming all variables remain unchanged, it would result in the generation of vectorized particles with nearly 100% homogeneity in the capsid libraries. The vector outputs usually consist of a mixture of different elements and protein units, all capable of enduring harsh conditions, and therefore outweighing the positive effects of Darwinian selection with unknown consequences when evaluated in receiving host. Thus, the current scalable AAV production workflow presents an opportunity for improvement, allowing for a better understanding of how capsid-engineered vectors influence VP stoichiometry and empty capsid formation [53].

**Fig. 2:**
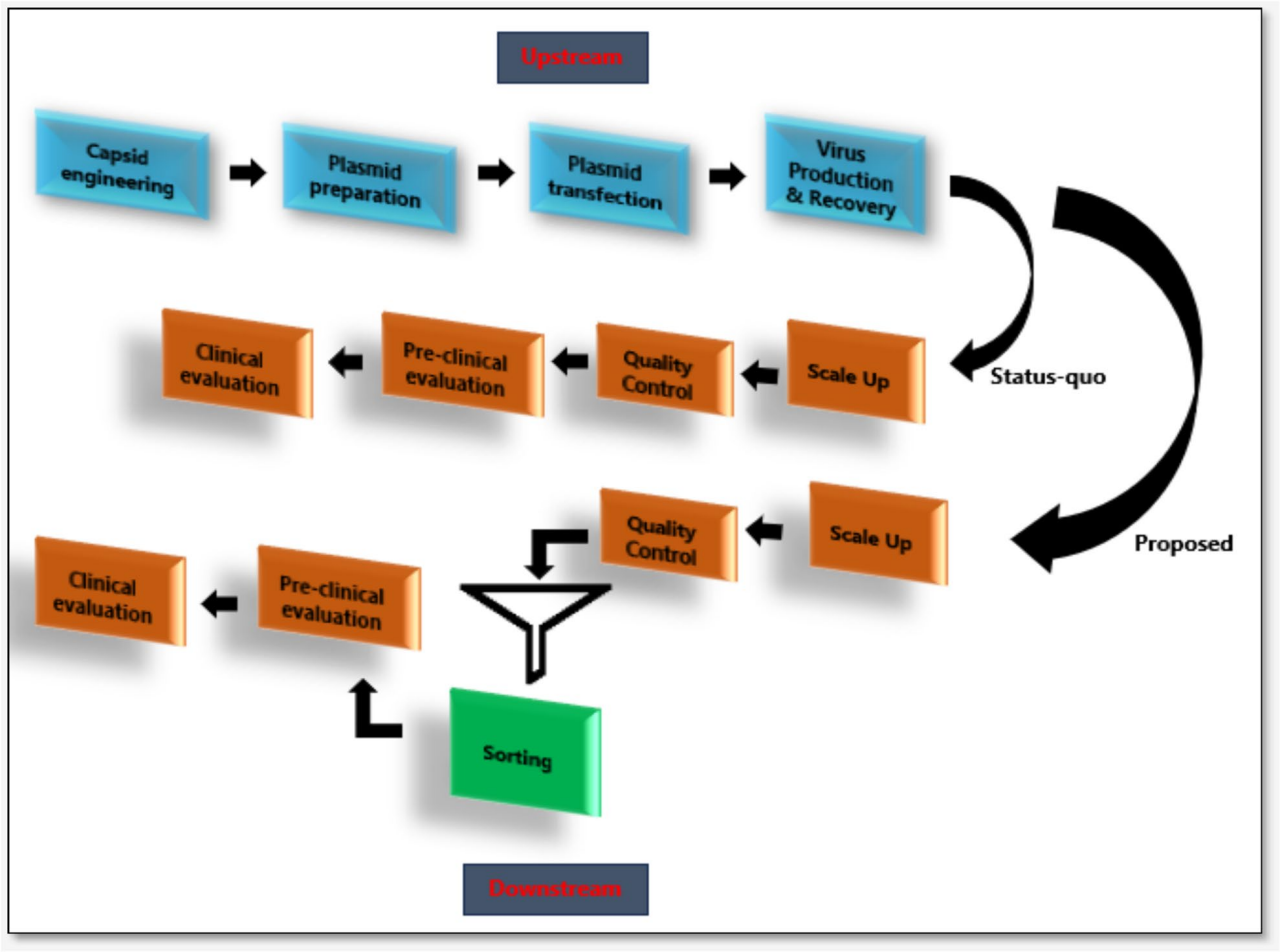
The status quo vs proposed preparation and evaluation of AAV-gene therapy products. The entire process can be sub-grouped into upstream and downstream. Upstream-Capsid modification: genetic or chemical manipulation of capsid gene sequences including preliminary process development; Plasmid preparation: the extraction and isolation of high quality viral plasmids; Plasmid transfection: triple transfection of viral plasmids in producing cells lines; Virus production and recovery: packaging and purification methods to isolate full and empty capsid vectors; Downstream-Scale-up: the expansion of production variables from small to medium or large scale; Quality control: current attempts to further refine AAV preparations and to eliminate empty capsid virions; Sorting: proposed analytical stage for categorisation of vectors based on the VP sequence homogeneity, molecular weight, transduction capabilities, buoyancy density and isoelectric point; Pre-Clinical and clinical evaluation: the testing procedures in in vivo models and humans in the clinic

Under normal circumstances, it is anticipated that AAV preparations like any other biological samples, to be contaminated with remnants of impurities such as unprocessed or host nucleic acids and protein fragments. Based on this understanding, off-the-shelf vectors may not necessarily represent the safest and most efficient clinically graded vectors. Even with advanced capsid engineering technologies, such as those developed by Dyno Therapeutics (bCap1), the transduction efficiency was < 30%, meaning that the fate of over 70% of the administered vectors is unknown. This requires the use of biological tools to identify and categorize not only empty capsid from full capsid vectors [54], but also those that share certain similarities, such as capsid protein sequences, modified VP stoichiometry ratios, transduction and infectious titres, and capsid-transgene cargo turnover rates, among others. Figure 2 summarises the AAV upstream and downstream manufacturing workflow with stepwise processes comparing the status quo to the recommended framework concept of sorting. Because current analytic methods are incapable of discriminating monomeric proteins or partially filled capsid from fully assembled 60-mer AAV particles, sorting of vectored particles based on unique characteristics is likely to abrogate the current research and development bottlenecks. With these tools in place, another advantage is the ability to administer uniform vectors containing uniform synthetic components to evade anti-AAV immune responses to ensure that vectors identified as outliers do not provoke unwanted immune response and clearance. Thresholding important characteristics including VP homogeneity, molecular weight, transduction capabilities, buoyancy density and isoelectric point can facilitate the categorization of vectors via automated systems [55–57].

## Data Availability

Not applicable.
